# Metabolome and Microbiome Signatures in the Leaves of Wild Tea Plant Resources Resistant to *Pestalotiopsis theae*

**DOI:** 10.3389/fmicb.2022.907962

**Published:** 2022-07-15

**Authors:** Yuqian Zhang, Jie Zhang, Changyu Yan, Meishan Fang, Lijie Wang, Yahui Huang, Feiyan Wang

**Affiliations:** ^1^College of Horticulture, South China Agricultural University, Guangzhou, China; ^2^Henan Key Laboratory of Tea Plant Comprehensive Utilization in South Henan, College of Tea Science, Xinyang Agriculture and Forestry University, Xinyang, China

**Keywords:** wild tea plant resource, *Camellia sinensis*, *Pestalotiopsis theae*, microbiome, metabolome

## Abstract

Tea (*Camellia sinensis*) is an important crop that is mainly used in the food industry. This study using the metabolome and microbiome investigates the resistance factors of wild tea plant resources against tea gray blight disease, which is caused by *Pestalotiopsis theae* (Sawada) Steyaert. According to the interaction analysis of tea leaves and pathogenic fungus, the resistance of wild tea plant resource “R1” (Resistance 1) to tea gray blight disease was significantly higher than that of wild tea plant resource “S1” (Susceptibility 1). The difference between “R1” and “S1” in the metabolome was obvious. There were 145 metabolites that significantly changed. The phenolic acids and flavonoids were the major increased categories in “R1,” and it included 4-O-glucosyl-sinapate and petunidin-3-o-(6”-o-p-coumaroyl) rutinoside. Six metabolic pathways were significantly enriched, including aminoacyl-tRNA biosynthesis, flavone, and flavonol biosynthesis. In terms of bacteria, there was no significant difference between “S1” and “R1” in the principal component analysis (PCA). *Pseudomonas* was the major bacterial genus in “S1” and “R1.” In addition, each of the two resources had its own predominant genus: *Cellvibirio* was a predominant bacterial genus in “S1” and *Candidatus_competibacter* was a predominant bacterial genus in “R1.” In terms of fungi, the fungal diversity and the abundance of the two tea plant resource samples could be distinguished clearly. The fungal component of “S1” was more abundant than that of “R1” at the genus level. *To*x*icocladosporium* was the predominant fungal genus of “S1,” and *Filobasidium* was the predominant fungal genus of “R1.” The relative abundance of *unclassified-norank-norank-Chloroplast* and *Penicillium* were significantly different between “S1” and “R1.” *Penicillium* was identified as a potential biomarker. They correlated with some metabolites enriched in “S1” or “R1,” such as L-arginine and quercetin-3-o-(2”-o-rhamnosyl) rutinoside-7-o-glucoside. Overall, phenolic acids, flavonoids, and *Penicillium* could be functional metabolites or microorganisms that contributed to improving the resistance of wild tea plant resources to tea gray blight disease.

## Introduction

Tea (*Camellia sinensis*) is an important crop that is mainly used in the food industry. Metabolites of polyphenols, alkaloids, theanine, tea saponin, and tea polysaccharides are rich in tea, and they have nutrients and functions in healthcare (Yang et al., 1999; Zheng et al., 2011). Tea gray blight disease, a destructive disease caused by the pathogen *Pestalotiopsis theae* (Sawada) Steyaert, can seriously harm tea leaves and the new shoots of the tea plant. Tea gray blight disease can greatly affect the yield of the tea product (Pallavi et al., 2012). The screening of tea plants' disease-resistance resources plays an important role in the tea industry. It is also important to study the disease-resistance mechanisms in tea.

Previous research has shown that tea plants contain many metabolites with obvious antibacterial properties, such as tea polyphenols and alkaloids (Karou et al., 2006; Ma et al., 2020). Primary and secondary metabolites compose a complex metabolic network in plants. Secondary metabolites can be used as biochemical barriers to resist pathogen invasion and can also be used as signal substances to participate in the transduction of plant-disease resistance response (Escobedo-Martínez et al., 2010; El-Hadidy and El-Ati, 2014). Secondary metabolites that are involved in the plant disease resistance process mainly include lignin, callose, and peroxidase (Luna et al., 2011; Schmelz et al., 2011; Ahuja et al., 2012; Sattler and Funnell-Harris, 2013; Yamane, 2013). Lignin, an important physical antimicrobial substance, causes lignification around the cell wall of the necrotic area to prevent the spread of pathogens (Qiao and Dixon, 2011; Sattler and Funnell-Harris, 2013). When the plant is infected by pathogens, callose can be deposited in a large number of plant cells, which is a response to biological stress such as pathogen invasion. Therefore, the accumulation of callose is an indicator of disease resistance in the study of plant disease resistance (Mt et al., 2003; Luna et al., 2011). Tea polyphenols have various biological activities, such as eliminating free radicals, and antioxidation (Liu et al., 2000). Caffeine also has an inhibitory effect on fungi such as *Hydromycetes, Rhizopus serpentonum*, and *Rhizopus piriocephalus* (Zhou et al., 2018; Thangaraj et al., 2020).

Microorganisms are important biological resources in nature and are ubiquitous in plants. Some microorganisms cannot cause disease symptoms when they survive in plant tissues. Some endophytic fungi can produce biochemical substances with insecticidal, antimicrobial, antitumor, immunosuppression, antioxidant, and other biological activities. Endophytic microorganisms can be positively involved in the growth and development of plants, for example, by promoting the ability to defend against biotic stresses. *B. subtilis* strain 330-2 produces lytic enzymes (laminarase, cellulase, and protease) that can degrade the cell walls of pathogenic fungi and inhibit the growth of *Rhizoctonia solani Kühn*. The leaf microbiome is very important for the biological control of leaf diseases (Bruisson et al., 2019; Becker et al., 2020). The special epiphytic bacteria around peach leaves have a great influence on the colonization of pathogenic bacteria (Randhawa, 1986). The endophytic fungus extracted from the leaves and stems of *Nyctanthes arbortristsi* can effectively inhibit pathogenic bacteria and fungus. Thus, these microbiomes have been regarded as potential biological resources to improve plant disease resistance. This present research on the metabolome and microbiome of the disease-resistant wild tea resources can further improve the theory of resistance in tea plants.

This study aimed to investigate the potential microorganisms and metabolites that contribute to disease resistance in tea plants via the metabolome and microbiome. The bacterial and fungal compositions of tea leaves were analyzed using 16S rRNA and ITS high-through sequencing, respectively. Additionally, the quadrupole time of flight mass spectrometry (UPLC–QTOF–MS) was applied to identify the metabolic profiles of the resistance and susceptibility of tea plant resources. The results of this research would provide directions for the mining of functional metabolites and microorganisms of tea tree resources against tea gray blight disease. They can also provide theoretical support for the prevention and control of tea gray blight disease, which would help guide the development of breeding tea plants with improved disease resistance.

## Materials and Methods

### Plant Materials

Fresh disease-free leaves from wild tea plant resources “S1” (Susceptibility 1), “R1” (Resistance 1), and “SR1” (Sub-resistance 1) were used for the analysis of disease resistance. The wild tea plant resources were collected in Yuntai Mountain, Anhua County, Yiyang City, Hunan Province, and non-artificial cultivation. “R1,” with obvious resistance to tea gray blight disease, and “S1,” with susceptibility to tea gray blight disease, were selected for microbiome and metabolome analysis. The tea leaves for the metabolome and microbiome were collected from nine tea plants, cleaned, and disinfected. This disinfection procedure ensures that there were no other microorganisms on the leaves' surface. The disinfection process was as follows: the leaves were washed 2-3 times with sterile water, soaked in 1% NaClO for 1 min, soaked in 75% alcohol for 1 min, and then washed 2-3 times with sterile water. Finally, the sterile water that was used for the last step of washing was cultured for microorganisms to determine whether the leaves' surface was thoroughly disinfected. Three tea plants were taken as one biological replicate, and three replicates of “S1” and “R1” were used for the microbiome and metabolome analysis.

### Resistance Phenotype to *Pestalotiopsis theae*

The third leaves of the three candidate tea plant resources were collected. After surface cleaning and disinfection, they were moisturized and placed in a plastic box. Fungal samples, 5 mm in diameter, were selected from the pathogenic fungus plate, cultivated for 5 days, and inoculated on the leaves. After inoculation for 24 h, the fungal samples were removed and the cultivation of leaves continued, which were observed for the formation of leaf spots. The diameters of the disease spots at different time points were measured with vernier calipers.

### Widely Targeted Metabolomics

The tea samples were freeze-dried with a vacuum freeze-dryer (Scientz-100F). The metabolite extraction method was based on Wang et al. (2020). The extracts were filtrated (SCAA-104, 0.22 μm pore size; ANPEL, Shanghai, China) before UPLC-MS/MS analysis.

For UPLC conditions and ESI-Q TRAP-MS/MS processing, the sample extracts were analyzed using the UPLC-ESI-MS/MS system (UPLC, SHIMADZU Nexera X2; MS, Applied Biosystems 4500 Q TRAP) equipped with a C18 column (Agilent SB-C18, 1.8 μm, 2.1 × 100 mm). The mobile phase consisted of solvent A, pure water with 0.1% formic acid, and solvent B, acetonitrile with 0.1% formic acid. The measurement procedure of the tea sample was based on Chen et al. (2013). Linear ion trap (LIT) and triple quadrupole (QQQ) scans were acquired on a triple quadrupole-linear ion trap mass spectrometer, AB4500 Q TRAP UPLC/MS/MS System, equipped with an ESI Turbo Ion-Spray interface, and controlled using Analyst 1.6.3 software (AB Sciex, Framingham, MA, USA). The ESI source operation parameters were based on Wang et al. (2020).

For UPLC-MS/MS baseline data analysis, unsupervised PCA was performed using the prcomp function in R. Data were assessed for unit variance prior to unsupervised PCA. The hierarchical cluster analysis (HCA) results of samples and metabolites were presented as heatmaps with dendrograms, while Pearson correlation coefficients (PCC) between samples were calculated using the cor-function in R and presented only as heatmaps. Significantly changed metabolites between the control (“S1”) and treatment (“R1”) groups were determined by VIP ≥ 1 and absolute Log_2_FC (fold change) ≥ 1. VIP values were derived from the OPLS-DA result, which also contains score plots and permutation plots. To avoid overfitting, a permutation test (200 permutations) was performed. The identified metabolites were annotated using the KEGG compound database (http://www.kegg.jp/kegg/compound/). The annotated metabolites were subsequently associated with the KEGG pathway database (https://www.kegg.jp/kegg/pathway.html).

### Microbiome Profile of Tea Leaf Samples

The genomic DNA of the microbial community was extracted from the tea leaf sample using FastDNA® Spin Kit for Soil (MP Biomedicals, USA) as directed by the manufacturer. The hypervariable region V5-V7 of the bacterial 16S rRNA gene and the fungal ITS gene were amplified with primer pairs 799F (5'-AACMGGATTAGATACCCKG-3') and 1193R (5'-ACGTCATCCCCAC CTTCC-3'), and ITS1F (5'-CTTGGTCATTTAGAGGAAGTAA-3') and ITS2R (5'-GCTGCGTTCTTC ATCGATGC-3'), respectively. The resulting PCR products of the 16S rRNA and ITS gene were extracted and purified. Following standard protocols, the Illumina MiSeq PE300 (Illumina, USA) platform was used for sequencing by Majorbio Bio-Pharm Technology Co. Ltd. (Shanghai, China). The sequenced results were analyzed through the Majorbio Cloud Platform (https://www.majorbio.com). The sequences were demultiplexed and quality-filtered using QIIME (version 1.9.1) with some criteria. The criteria reference was Zhang et al. (2018). The bioinformatics procedure is as follows: in-house Perl scripts were used to analyze the alpha- and beta-diversity. Ribosomal Database Project (RDP) was used to annotate the taxonomic information from OTUs (Cole et al., 2014). Significant differences between the two groups of samples were evaluated using the paired Student's *t*-test in the a-diversity indices. Taxonomical and functional differences between the two groups of samples were analyzed using the STAMP 2.1.3 software (Parks et al., 2014), and the *p*-values were calculated via Welch's *t*-test. LEfSe analysis was performed to identify the most differentially abundant taxa between the two groups of samples (Segata et al., 2011), and the most discriminant taxa were distinguished by linear discriminant analysis (LDA) score > 2 and *p* < 0.05. Differences in relative taxa abundance between the two groups of samples were calculated using a non-parametric Wilcoxon rank-sum test. Taxa-relative abundance in the difference between the two groups of samples was calculated by a non-parametric Wilcoxon rank-sum test. Population differences were analyzed with a unidirectional ANOVA. *p* < 0.05 was deemed statistically significant.

### Statistical Analysis

All statistical analyses have been carried out using SPSS 22 statistics. The differences between the control (“S1”) and treatment groups (“R1”) were analyzed using the Student *t*-tests. Statistical significance was assessed by *p* < 0.05. Correlations between the microbiotas and metabolites were analyzed by Spearman correlation analysis.

## Results

### Resistance Phenotypes to *Pestalotiopsis theae*

**Figure 1:**
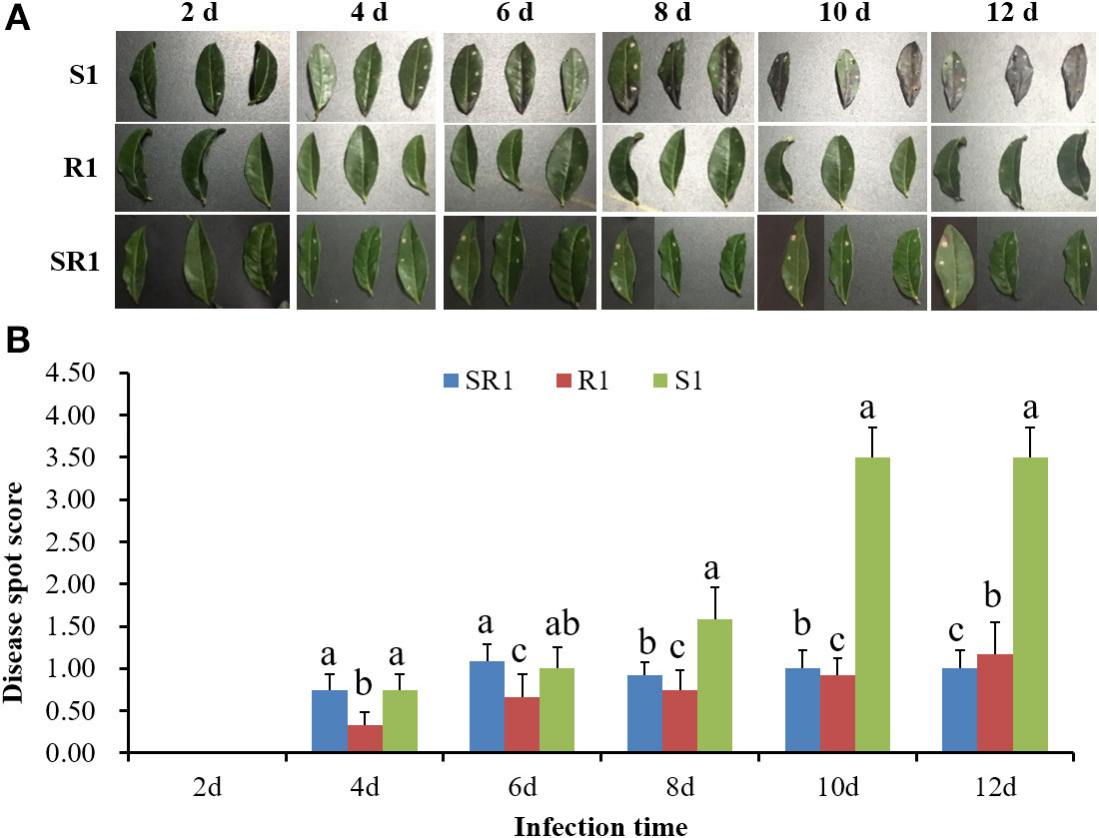
**(A)** Analysis of the interaction between wild tea leaves and tea gray blight pathogen. **(B)** Disease resistance analysis of wild tea plant resources and tea gray blight pathogen. S1, Susceptibility 1; R1, Resistance 1; SR1, Sub-resistance 1. Different lowercase letters in the histogram indicate significant difference of disease spot score among “S1”, “SR1”, and “R1” at the same time of infection (*p* < 0.05, SNK-q test).

The interaction analysis results of the third tea leaves and the tea gray blight pathogen showed that the wild tea plant resource “R1” had obvious resistance to tea gray blight disease, and no obvious disease spots were found in the leaves during the whole infection cycle (Figure 1A). Tea plant resource “S1” was sensitive to tea gray blight disease. On the 6th day of infection, the leaves showed obvious browning (Figure 1). On the 12th day, the “S1” leaves were dead (Figure 1). The resistance of the tea plant resource “SR1” to tea gray blight disease was between those of “S1” and “R1” (Figure 1). Consequently, “S1” and “R1” were selected for subsequent analysis of the microbiome and metabolome to obtain differences in microbial diversity and metabolites between “S1” and “R1.”

### Metabolism Differences Between the Two Wild Resources

The tea leaf samples were analyzed using UHPLC-MS/MS in positive and negative modes (Supplementary Figure 1A). A typical TIC plot of one QC sample and the multipeak detection plot of metabolites in the multiple reaction monitoring (MRM) modes were illustrated in Supplementary Figures 1B,C. The TIC plot represents a continuous description of the intensity sum of all ions in the mass spectrum at different time points. The multipeak detection plot of metabolites in the MRM mode revealed the substances that were detected in the samples. In the multipeak detection plot, each peak in a different color represents a detected metabolite. Information on metabolite serial numbers, peak integration values, and metabolite names are listed in Supplementary Table 1.

**Figure 2:**
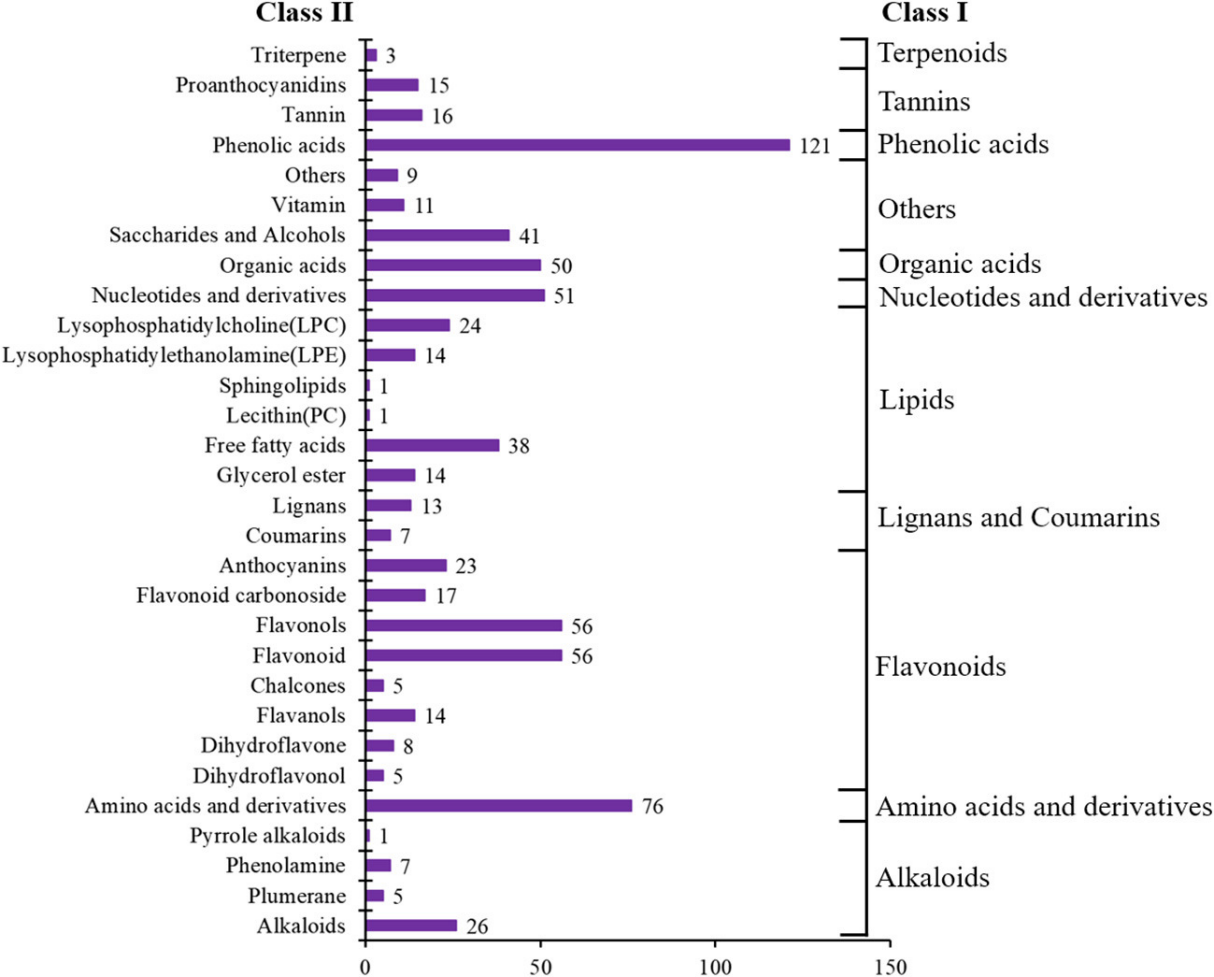
Number of metabolite categories identified in all samples.

**Figure 3:**
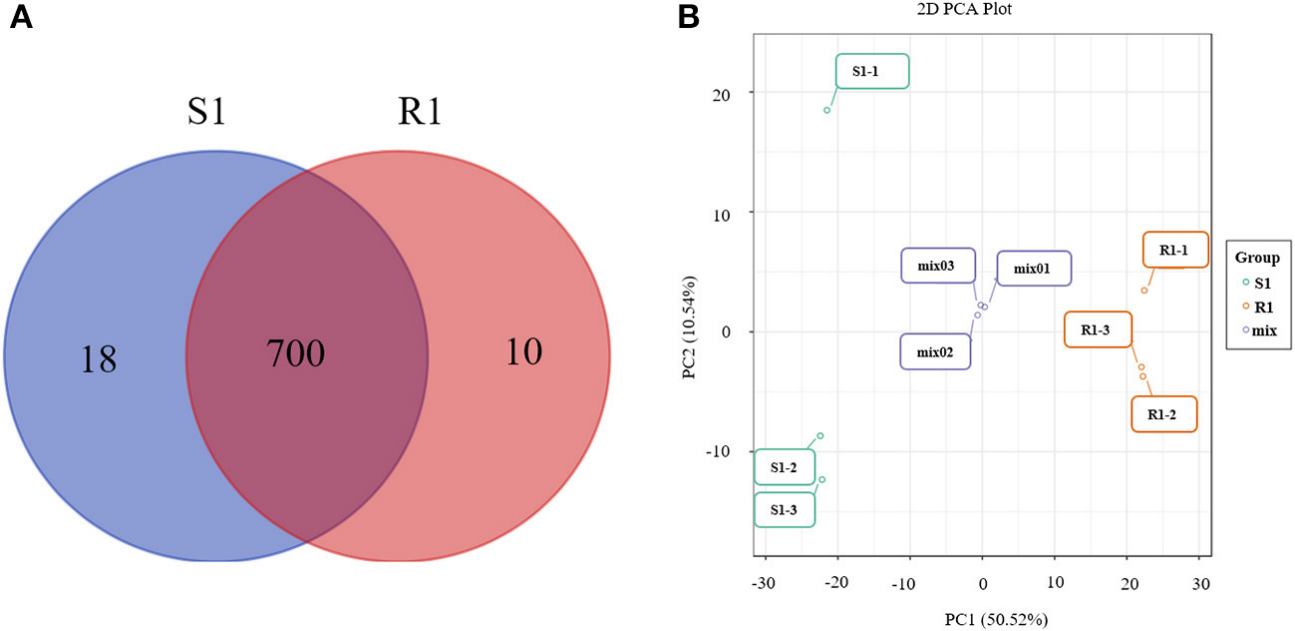
Venn diagrams of metabolites detected in each group **(A)** and principal component analysis (PCA) score plot for mass spectrum data of tea samples and quality control samples **(B)**. The *X*-axis represents the first principal component (PC1) and the *Y*-axis represents the second principal component (PC2) in the PCA score plot.

In total, 728 metabolites were identified, including 184 flavonoids, 121 phenolic acids, 92 lipids, 76 amino acids and their derivatives, 51 nucleotides and their derivatives, 50 organic acids, 39 alkaloids, 31 tannins, 20 lignans and coumarins, 3 terpenoids, and 61 others (Figure 2, Supplementary Table 1). The metabolites of class II belong to class I in Figure 2. All metabolites were analyzed by ANOVA (Supplementary Table 1). As shown in the Venn plot, the most detected and identified metabolites can be found in the two tea leaf samples (Figure 3A).

The quality control samples (QC) were prepared by mixing extracts from two tea leaf samples. As shown in the principal component analysis (PCA) plots, there was a large separation of trends between the “S1” and “R1” leaves and little intragroup variation was observed (PC1=50.52% and PC2=10.54%, Figure 3B). OPLS-DA modeling can maximize the difference between different groups and can be performed to evaluate the data quality and identify potential biomarkers. As is shown, the values of R^2^Y (0.999) were high and the values of Q^2^ were >0.5 (Supplementary Figure 2A), indicating good reliability and predictability of the model. In the permutation test of the OPLS-DA, the sequential order of categorical variable Y was randomly changed many times (n=200) (Supplementary Figure 2B). Three corresponding OPLS-DA models were established on each occasion (Supplementary Figures 2C,D). Thus, the R^2^ and Q^2^ values of the stochastic models were obtained. The results suggested that these methods were of great importance for preventing the test model from overfitting and for evaluating the statistical significance of the model. As shown in Supplementary Figure 2B, the original model's R^2^Y values were close to 1 in “S1” and “R1,” respectively. The results indicate that the established models were an accurate description of the real situation in all sample data. The Q^2^ values in “S1” and “R1” were close to 1 (0.966), and these show that a similar distribution would be generated if other new samples were added to the models. As a whole, the original model of the “S1” and “R1” groups can ideally explain the difference between each treatment group and the control group. Furthermore, there was a gradual decrease in the retention degree of the permutation. The R^2^Y' and Q^2^' values of permutation models are less than those of the R^2^Y and Q^2^ of the stochastic model shown in Supplementary Figure 2B. Therefore, these results indicate that the PCA and OPLS-DA models in the present study demonstrate good repeatability and reliability. The potential biomarkers were screened according to the VIP value (VIP > 1), the absolute value of fold change ≥ 2, and statistical tests *p* < 0.05.

**Figure 4:**
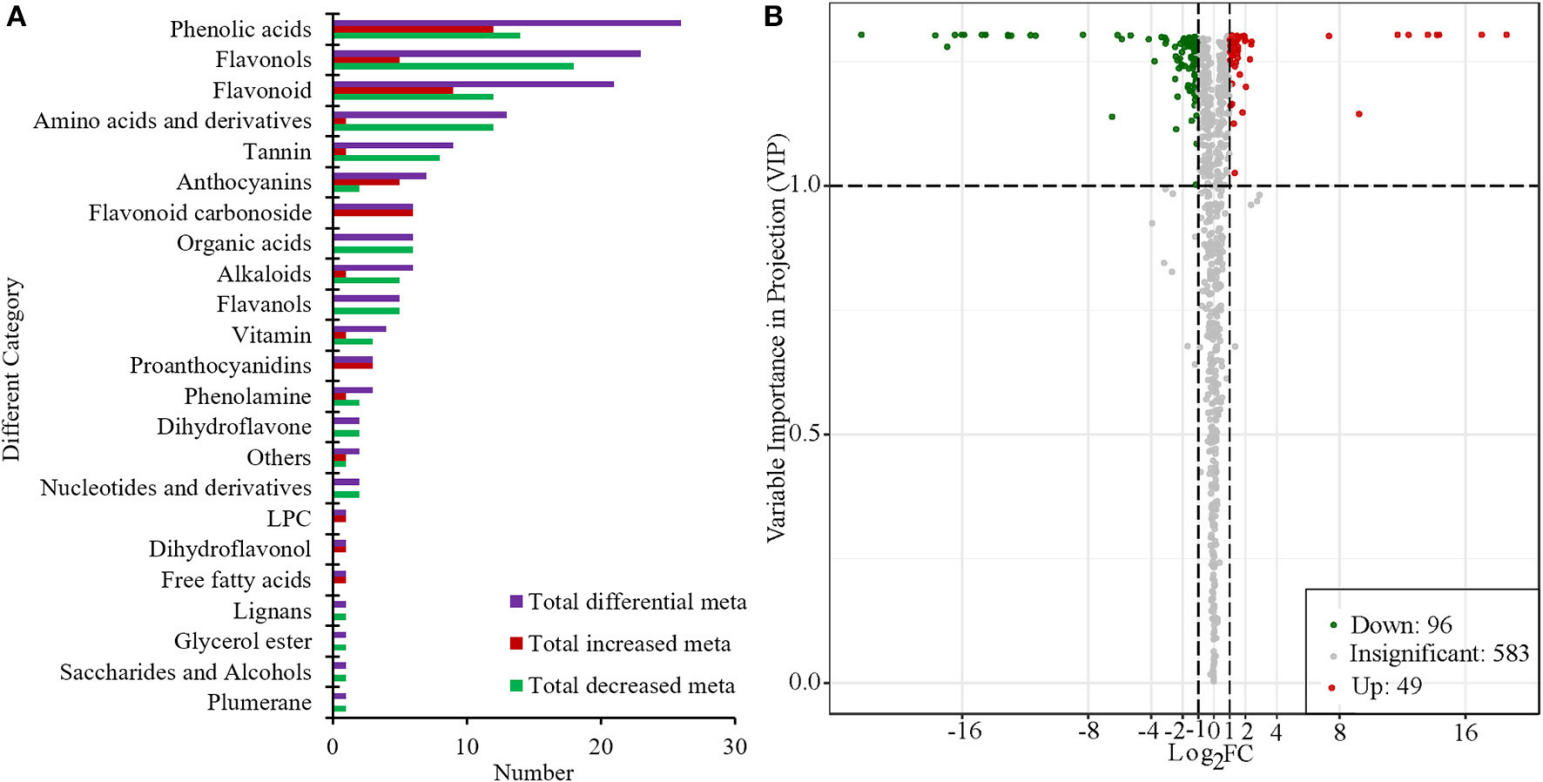
Number of different categories **(A)** and volcano plot of metabolites **(B)** in “R1” vs. “S1.”

Based on the PCA and OPLS-DA results, the VIP can be used for the preliminary screening of different metabolites between the treatment and the control groups. Moreover, differential metabolites can be further screened by combining the VIP value with the fold-change value. In this study, there were 145 metabolites that significantly changed (|Log_2_Foldchange| ≥ 1 and VIP > 1). Among them, compared with “S1,” 96 metabolites were significantly decreased and 49 metabolites were significantly increased in “R1” (Figure 4, Supplementary Table 2). Flavonols, phenolic acids, amino acids and their derivatives, and flavonoids were the main categories of significantly changed metabolites (SCMs). The most increased categories were phenolic acids and flavonoids in “S1” (Figure 4A). Among all the differentially enriched metabolites, 21 metabolites were particularly obvious, such as quercetin-7-o-rutinoside-4'-o-glucoside and petunidin-3-o-(6”-o-p-coumaroyl) rutinoside (Supplementary Figure 3).

The potential biomarkers were used for pathway analysis using KEGG topology analysis. In this study, 145 SCMs were used for KEGG analysis. The differential metabolites were classified into corresponding pathways according to the information from the pathway database (Supplementary Table 3). The pathway analysis of SCMs involved a total of 52 pathways (Supplementary Figures 4, 5). Six metabolic pathways (rich factor value > 0, *p* < 0.05) were significantly enriched, including aminoacyl-tRNA biosynthesis (rich factor value = 0.56); glucosinolate biosynthesis (rich factor value = 0.75); tropane, piperidine, and pyridine alkaloid biosynthesis (rich factor value = 0.67); 2-oxocarboxylic acid metabolism (rich factor value = 0.39); flavone and flavonol biosynthesis (rich factor value = 0.42); and valine, leucine, and isoleucine degradation (Rich factor value = 0.50). It should be noted that aminoacyl-tRNA biosynthesis, 2-oxocarboxylic acid metabolism, and flavone and flavonol biosynthesis encompass a large portion of the total metabolites in “S1” and “R1” (Supplementary Figure 5).

### Microbial Difference Analysis in Tea Leaves

The bacterial and fungal compositions of “S1” and “R1” leaves were analyzed using 16S rRNA and ITS high-throughput sequencing, respectively. A total of 69,996 raw reads of 16S rRNA and 4,06,834 raw reads of ITS were obtained from six tea leaves samples (*n* = 3 for each group) with an average of 11,666 reads of bacterial and 67,805 reads of fungi per sample. The average read lengths of 16S rRNA and ITS were 377.18 bp and 232.51 bp, respectively. A total of 207 distinct operational taxonomic units (OTUs) of bacteria and 191 OTUs of fungi were observed in all samples. The Simpson index and Sobs index of bacteria and fungi did not show a significant difference between “S1” and “R1” (Supplementary Figures 6A,B). Genus-based principal component analysis (PCA) showed distinct fungal profiles between “S1” and “R1” (Supplementary Figure 7A), but the PCA of bacteria did not show distinct bacterial profiles between “S1” and “R1” (Supplementary Figure 6C). Also, the results of the hierarchical clustering of fungus and bacteria did not show better separations (Supplementary Figures 6D, 7B).

In Supplementary Figure 6E, *Proteobacteria* and *Actinobacteria* dominated the leaf microbiota composition at the phylum level of bacteria, contributing to 92 and 7.1% of the leaves' microbiota in “S1,” and 91 and 8% of the leaves' microbiota in “R1,” respectively. At the genus level, the relative abundance of microbes showed a difference between “S1” and “R1” (Supplementary Figure 6F). In Supplementary Figure 7C, *Ascomycota* and *Basidiomycota* dominated the leaf microbiota composition at the phylum level of fungi, contributing to 83 and 17% of the microbiota in “S1,” and 70 and 30% of the microbiota in “R1,” respectively. At the genus level, the relative abundance of fungi showed the difference between “S1” and “R1” (Supplementary Figure 7D). The differences in leaf microbiota among these groups were also evidenced by linear discriminant analysis effect size (LEfSe), which showed the most different abundant taxon of two tea plant resource samples (Supplementary Figures 8, 9). Using a metagenomic biomarker discovery approach, *Cyanobacteria* was found to be significantly enriched in the “S1” samples (LDA score > 3), while *Burkholderia–Caballeronia–Paraburkholderia* (LDA score > 3.5) and *Penicillium, Plectosphaerella*, and *Plectosphaerellaceae* (LDA score > 4) were identified as biomarkers of the “R1” group (Supplementary Figures 8, 9).

At the genus level of bacteria, 151 genera coexisted in the two tea resources. A total of 40 genera only existed in the “S1” resources and 29 genera only existed in the “R1” resources (Supplementary Figure 10A). *Pseudomonas* was the predominant genus, contributing to 79.74% of the common microbiota in the two tea samples (Supplementary Figure 11A). *Cellvibirio* was the predominant genus (10.82%) only in “S1,” and *Candidatus_competibacter* was the predominant genus (28.90%) only in “R1” (Supplementary Figures 11B,C).

At the genus level of fungi, 102 genera coexisted in the two tea resources. A total of 41 genera only existed in the “S1” resources and 27 genera existed in the “R1” resources (Supplementary Figure 10B). *Cutaneotrichosporon* was the predominant genus, contributing to 23.12% of the common microbiota in the two sample groups (Supplementary Figure 12A). *Toxicocladosporium* was the predominant genus (18.85%) only in “S1” and *Filobasidium* was the predominant genus (13.30%) only in “R1” (Supplementary Figures 12B,C). The results show that the microbiota composition at the genus level of “S1” is more abundant than that in “R1.”

**Figure 5:**
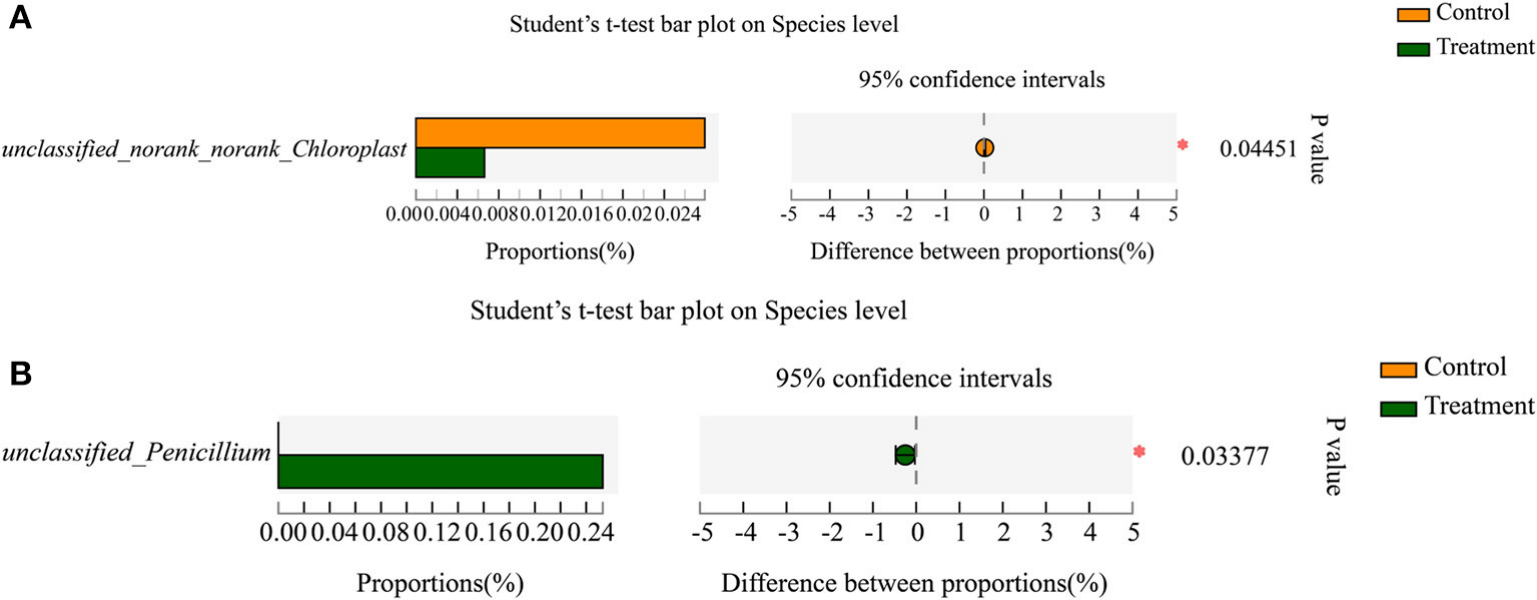
The significantly enriched bacterial and fungal species in “S1” and “R1” samples. **(A)** The significantly enriched bacterial species. **(B)** The significantly enriched fungal species.

*Norank_norank_Chloroplast* (*p*=0.04451) was a significantly different enrichment bacterial genera between the two tea resources (Supplementary Figure 13). *Penicillium* (*p* = 0.02156) was a significantly different enrichment fungal genera between the two tea resources (Supplementary Figure 14). At the species level of bacteria, the relative abundance of *unclassified_norank_norank_ Chloroplast* (*p* = 0.04451) showed a significant difference between the two tea resources (Figure 5A). At the species level of fungi, the relative abundance of *unclassified*_*Penicillium* (*p* = 0.03377) showed a significant difference between the two tea samples (Figure 5B).

### Correlations Between Microorganisms and Metabolites in Tea Leaves

Correlation analysis between the leaves” microorganisms and metabolites indicates potential mutual contributions. To investigate the contributions of metabolites to leaves' microbiota, we performed RDA on the significantly changed metabolites related to the leaves' microbiota (Supplementary Figure 15). The top three high-enrichment or low-enrichment metabolites contributed to the leaves' microbiota. We found that the top three high-enrichment metabolites, namely quercetin-7-o-rutinoside-4'-o-glucoside, 2,3-di-o-galloyl-d-glucose, and L-arginine contributed to the bacteria and fungi of “S1” leaves. However, the top three low-enrichment metabolites, namely luteolin-6-c-glucoside-7-o-(6”-p-coumaroyl) glucoside, isovitexin-2”-o-(6”'-p-coumaroyl) glucoside, and vitexin-7-o-(6”-p-coumaroyl) glucoside contributed to bacteria and fungus of “R1” leaves (Supplementary Figure 15).

In addition, the metabolites, and the bacterial and fungal genera in two tea resources were used for Spearman's correlation analysis. In Supplementary Figures 16, 17, the relative abundance of *unclassified_Gammaproteobacteria, Microbacterium, norank_norank_ Gaiellales, Aeromonas, Bacillus, norank_norank_Chloroplast, Pseudomonas, Ralstonia, unclassified_Enterobacteriaceae, Sphingomonas* and *Penicillium, Pseudocercospora, Plectosphaerella, Cladosporium, unclassified_Ascomycota, Phyllosticta, Strelitziana, Toxicocladosporium, Bipolaris, Trichoderma*, and *unclassified_Xylariales* were significantly correlated with the top twenty-one metabolites enriched in “S1” or “R1.” The positive and negative correlations were as follows: *Microbacterium, norank_norank_Gaiellales, Aeromona*, and *unclassified-norank-norank-Chloroplast* were significantly positively correlated with the levels of delphinidin-3-o-(2”'-o-p-coumaroyl) rutinoside and isorhamnetin-3-o-rutinoside-7-o-rhamnoside, but were significantly negatively correlated with some SCMs, such as 1,2,3-tri-o-galloyl-d-glucose and castanoside. *Bacillus, norank_norank_Chloroplast, Pseudomonas, Ralstonia, unclassified_ Enterobacteriaceae*, and *Sphingomonas* were significantly positively correlated with the levels of some SCMs, such as 2,3-tri-o-galloyl-d-glucose, 2,3-di-o-galloyl-d-glucose, dicaffeoylquinic acid-o-glucoside, kaempferol-3-o-(2”-p-coumaroyl) galactoside, feruloylmalic acid, feruloyltartaric acid (fertaric acid), and p-coumaroylmalic acid, but were significantly negatively correlated with delphinidin-3-o-(2”'-o-p-coumaroyl)rutinoside, quercetin-3-o-(2”-o-rhamnosyl)rutinoside-7-o-glucoside, petunidin-3-o-(6”-o-p-coumaroyl) rutinoside, isorhamnetin-3-o-rutinoside-7-o-rhamnoside, luteolin-7-o-sophoroside-5-o-arabinoside, etc. *Penicillium, Pseudocercospora*, and *Plectosphaerella* were significantly positively correlated with the levels of delphinidin-3-o- (2”'-o-p-coumaroyl) rutinoside, quercetin-3-o- (2”-o-rhamnosyl) rutinoside-7-o-glucoside, petunidin-3-o-(6”-o-p-coumaroyl) rutinoside, and isorhamnetin-3-o-rutinoside-7-o-rhamnoside but were significantly negatively correlated with some metabolites such as L-arginine, eriodictyol-7-o-glucoside, cyanidin-3-o- (6”-o-p-coumaroyl) rutinoside-5-o-glucoside, and 6-c-methylquercetin-3-o-rutinoside. *Strelitziana, Toxicocladosporium, Bipolaris, Trichoderma*, and *unclassified_Xylariales* were significantly positively correlated with the levels of L-arginine and eriodictyol-7-o-glucoside. *Trichoderma* and *unclassified_Xylariales* were significantly negatively correlated with delphinidin-3-o-(2”'-o-p-coumaroyl) rutinoside.

## Discussion

Although some secondary metabolites, such as lipids (Lim et al., 2017), flavonoids (Cushnie and Lamb, 2011), alkaloids (Yang et al., 2016), polyphenols (Bazzaz et al., 2016), and glycoside (Escobedo-Martínez et al., 2010), are not directly involved in plant growth and development, they can nonetheless act as a chemical defense. That is, they can be used for defending against the damage caused by plant diseases in specific environments and physiological conditions. Flavonoids, as one of the substances with defense functions, are widely found in plants. In maize, 2,4-dihydroxy-7-methoxy-2H-1,4- benzoxazin-3(4H)-one (DIMBOA) can resist fungus (Frey et al., 1997). The isoflavones also have antimicrobial functions (Undhad et al., 2021). Research has shown that under the stimulation of pathogenic bacterial infection, tea leaves can increase polyphenols as antibacterial factors, and the cortex of roots and stems can also produce antitoxin (Turkmen et al., 2007).

In the present study, the tea plant resource “R1” had obvious resistance to tea gray blight disease, but “S1” was susceptible. Flavonoids, phenolic acids, amino acids, and derivatives were the major differential accumulated categories of SCMs. The major SCMs include kaempferol, luteolin, vitexin, quercetin, and their glycosides. “R1” sample specifically enriched petunidin, luteolin, vitexin, isovitexin, quercetin, and isorhamnetin's glycoside. In *Arabidopsis*, quercetin can protect *Arabidopsis* against *Pseudomonas syringae* pv. Tomato DC3000 infection through both quercetin-mediated H_2_O_2_ generation and the involvement of SA and NPR1 (Jia et al., 2010). Isovitexin is a biologically active flavone C-glycosylated derivative of apigenin that possesses anti-inflammatory and antioxidant properties in vegetables and fruits (Zielińska-Pisklak et al., 2015; Lv et al., 2016). Isorhamnetin augments the cellular antioxidant defense capacity by activating the Nrf2/HO-1 pathway (Hyun, 2016). Sinapate helps to improve pathogen resistance for *Botrytis cinerea* by UV-B radiation in *Arabidopsis* (Demkura and Ballaré, 2012). Vitexin has been reported to have inhibitory effects on Gram-negative bacteria, especially *Pseudomonas aeruginosa* and *Bordetella petrii* (Das et al., 2016; Rath et al., 2016; Corrêa et al., 2021), and vitexin's glucoside has a significant antioxidant effect (Fang et al., 2016; Wu et al., 2021). Thus, the enriched metabolites in the “R1” sample may contribute to the disease resistance to tea gray blight disease. These metabolites therefore provided the research directions for analyzing the function of metabolites through the interaction between metabolites and pathogens.

The dominant species and compositions of antimicrobial bacteria are closely related to plants' resistance to disease (Mendes et al., 2011; Lareen et al., 2016). Bacteria can have wide-ranging effects on the host plants. For example, they can affect many plant processes such as disease resistance, drought tolerance, life cycle phenology, and overall vigor (Panke-Buisse et al., 2017). The research on the microorganisms and *Cassava* phenotype shows that *Lactococcus* sp., *Pantoea dispersa*, and *Saccharomyces cerevisiae* are closely associated with cassava disease resistance (Bonito et al., 2014). An antagonistic function has been reported for the pathogen *Pantoea dispersa*, an indigenous endophytic bacterium of rice seeds that can produce auxins to inhibit the pathogen *Fusarium oxysporum*, allowing it to play an important role in the defense against pathogens (Verma et al., 2017). *Pantoea dispersa* has fungicidal properties and was found to significantly inhibit the growth of *Ceratocytis fimbriata* on the leaves and tuberous roots of a susceptible sweet potato cultivar (Jiang et al., 2019). Gu et al. (2020) found that the modulation of siderophore plays an important role in iron competition and biocontrol of the soil-borne pathogen *Ralstonia solanacearum*. In our study, *Proteobacteria, Actinobacteria, Ascomycota*, and *Basidiomycota* dominated the microbiota composition at the phylum level. At the genus level, *Pseudomonas* and *Cutaneotrichosporon* were the predominant genera in the two sample groups. *Cellvibirio, Toxicocladosporium* or *Candidatus_competibacter*, and *Filobasidium* were the predominant genera only in “S1” or “R1.” *Cellvibrio* has been isolated from soil environments; nevertheless, strains belonging to the genus have also been found in aquatic environments (Rhee et al., 2010). The *Cellvibrio* genus, especially *C. japonicus*, has been recognized and studied for its industrial enzymic potential to degrade a variety of polysaccharides, including those present in the plant cell wall (DeBoy et al., 2008). This means that the members of *Cellvibrio* might hurt plant cells. The yeast *Filobasidium* has been described as an important member of the wine consortia for its role in creating specific flavor profiles or reducing final alcohol content (Cureau et al., 2021). At the species level, the relative abundance of *unclassified_norank_norank_Chloroplast* and *unclassified_Penicillium* have shown significant differences between the two groups of samples. Furthermore, we found that the proportional abundance of *Penicillium* in the “R1” group was particularly significantly increased. A high level of *Penicillium* was identified as a potential biomarker of the “R1” group via LEfSe analysis. According to the correlation research between metabolites and microorganisms, the predominant genera, such as *norank_norank_Chloroplast, Pseudomonas, Penicillium*, and *Toxicocladosporium* are significantly correlated with the top SCMs enriched in “S1” or “R1,” including L-arginine, castanoside B, cyanidin-3-o-(6”-o-p-coumaroyl) rutinoside-5-o-glucoside, dicaffeoylquinic acid-o-glucoside, isorhamnetin-3-o-rutinoside-7-o-rhamnoside, luteolin-7-o-sophoroside-5-o-arabinoside, p-coumaroylmalic acid, and quercetin-3-o-(2”-o-rhamnosyl) rutinoside-7-o-glucoside.

Overall, the present study demonstrated an obvious difference between “S1” and “R1” tea plant resources in their metabolomes. The phenolic acids and flavonoids were the categories in “R1” samples with major increases and they included 4-O-glucosyl-sinapate and petunidin-3-o-(6”-o-p-coumaroyl) rutinoside. The major bacterial genus was *Pseudomonas* in “S1” and “R1.” *Cellvibirio* was predominant genus in “S1” and *Candidatus_competibacter* was predominant genus in “R1.” The microbiota of “S1” is more abundant than that in “R1.” *Unclassified_norank_norank_Chloroplast* and *unclassified_Penicillium* showed significant differences between “S1” and “R1.” *Penicillium* was identified as a potential biomarker. They were both significantly correlated with some metabolites. Phenolic acids and flavonoids, as well as *Penicillium* can be functional metabolites and microorganisms that may contribute to tea plants' resistance to tea gray blight disease. These will be used for further research on the mechanism of disease resistance.

## Data Availability Statement

The datasets presented in this study can be found in online repositories. The names of the repository/repositories and accession number(s) can be found below: NCBI BioProject - PRJNA823872.

## Author Contributions

YZ and JZ designed and performed the experiments and wrote the manuscript. MF and LW revised the manuscript. YH provided the wild tea plant resources, participated in sample preprocessing, and part of the manuscript writing. FW conceived the experiments and revised the manuscript. All authors contributed to the article and approved the submitted version.

## Funding

This study was funded by the National Natural Science Foundation of China (No. 31902077), the Open Fund of Henan Key Laboratory of Tea Comprehensive Utilization in South Henan (No. HNKLTOF2020007), and the Guangdong Provincial Special Fund for Modern Agriculture Industry Technology Innovation Teams (No. 2021KJ120). The funders were not involved in the design of the study, collection, analysis, interpretation of data, and manuscript writing.

## Conflict of Interest

The authors declare that the research was conducted in the absence of any commercial or financial relationships that could be construed as a potential conflict of interest.

## Publisher's Note

All claims expressed in this article are solely those of the authors and do not necessarily represent those of their affiliated organizations, or those of the publisher, the editors and the reviewers. Any product that may be evaluated in this article, or claim that may be made by its manufacturer, is not guaranteed or endorsed by the publisher.
