# Supplementary material for: Metabolome and Microbiome Signatures in the Leaves of Wild Tea Plant Resources Resistant to Pestalotiopsis theae
Source: Front Microbiol. 2022 Jul 15;13:907962. doi: 10.3389/fmicb.2022.907962 (PMC9335280; doi:10.3389/fmicb.2022.907962)
Supplement: Supplementary file 1 [file Presentation_1.pdf]

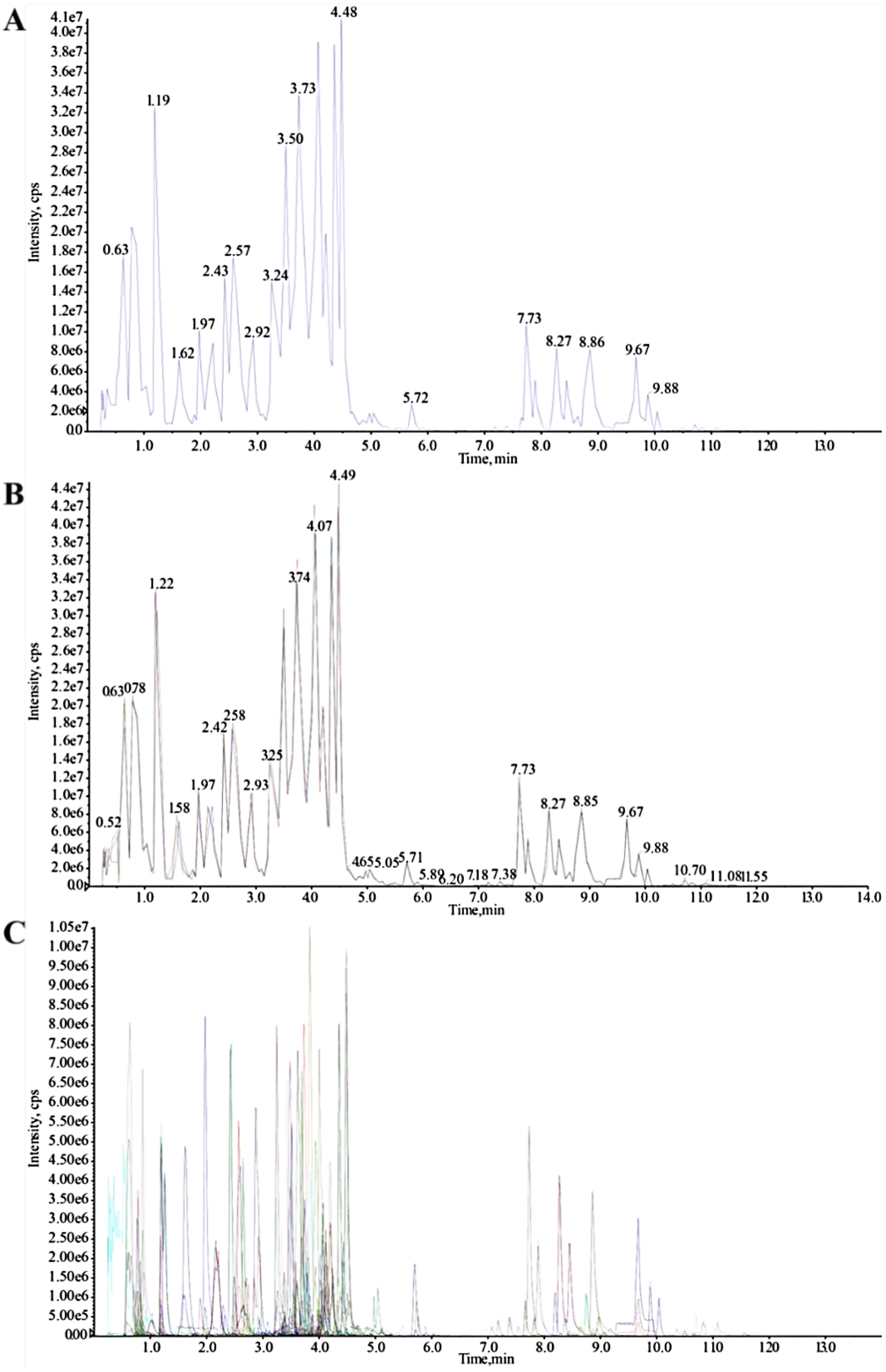

Supplementary Figure 1. Total ion current of one quality control sample by mass spectrometry detection (A) and total ions current overlaps of the three quality control samples by mass spectrometry detection (B) and multi-peak detection plot of metabolites in the multiple reaction monitoring mode (C).

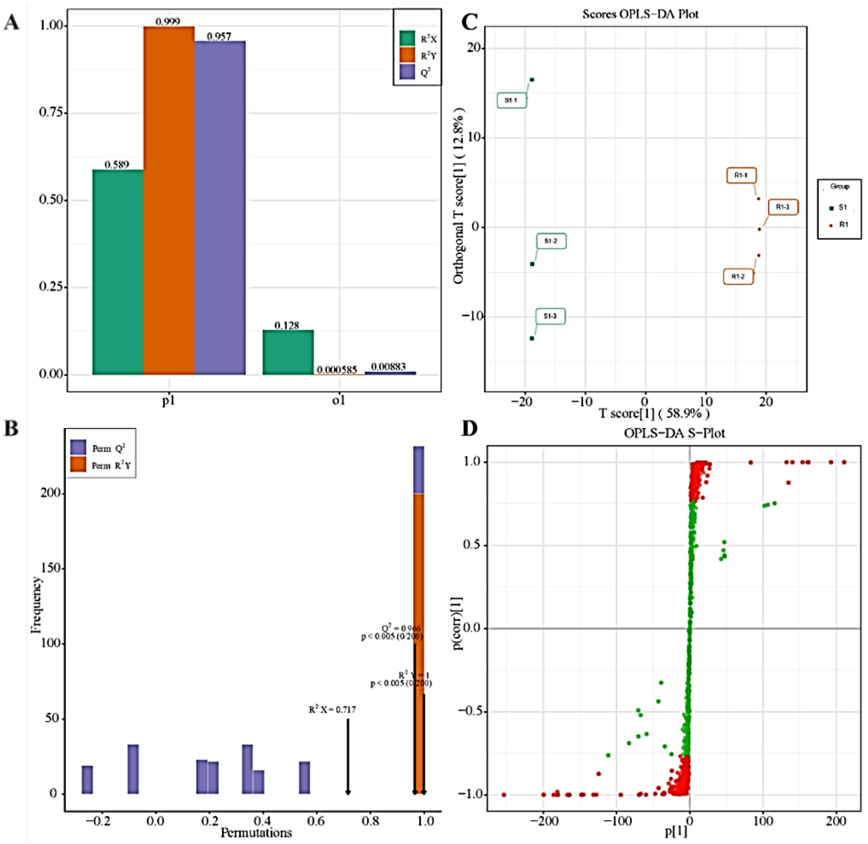

Supplementary Figure 2. Orthogonal projections to latent structures-discriminant analysis (OPLS-DA) results. (A) Score scatter plot of the OPLS-DA model for the comparison. (B) Permutation test of the OPLS-DA model for the comparison. (C) OPLS-DA-plot for the comparison. (D) OPLS-DA-S-plot for the comparison.

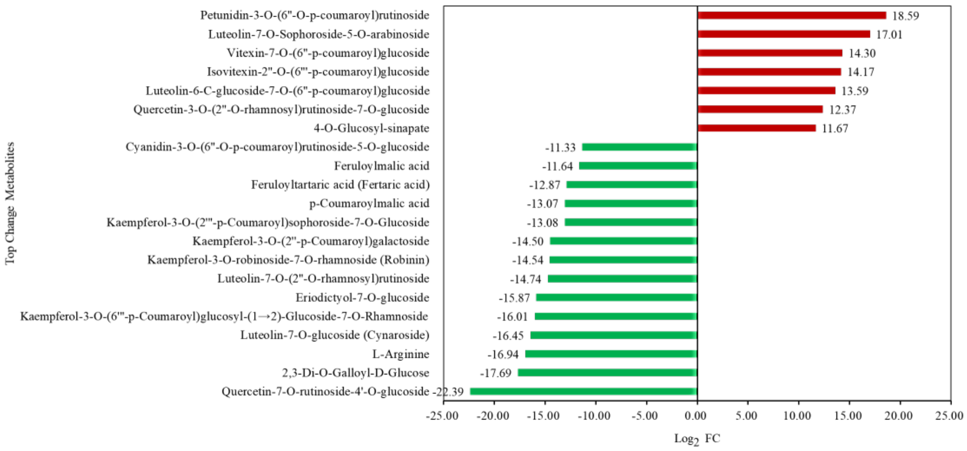

Supplementary Figure 3. Top significantly change metabolites in ‘R1’ *vs.* ‘S1’

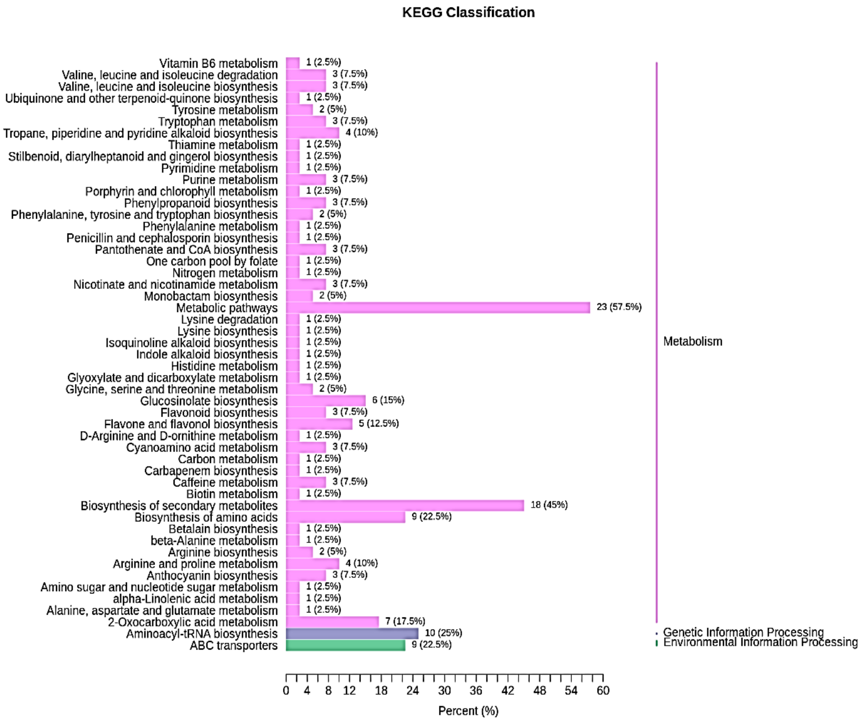

Supplementary Figure 4. KEGG pathway analysis of differential metabolites

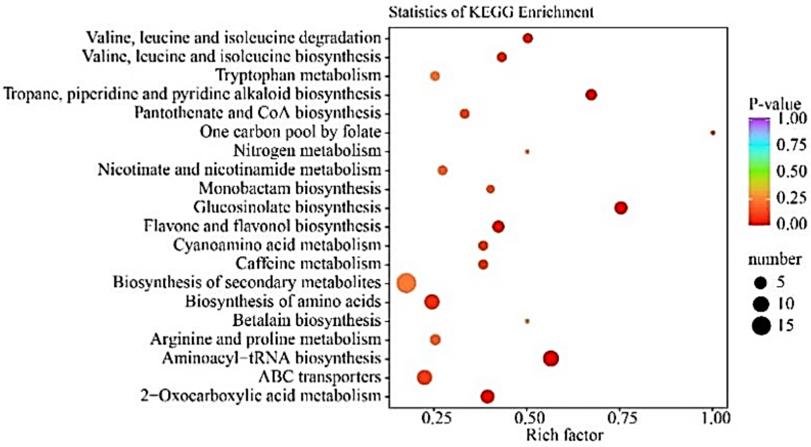

Supplementary Figure 5. KEGG pathway analysis of differential metabolites.

Each bubble in the plot represents one metabolic pathway whose abscissa and bubble size indicate the magnitude of the rich factors and metabolites number of the pathway in the topological analysis, respectively. Bubble colors represent the *P*-values of enrichment analysis, with darker colors showing a higher degree of enrichment.

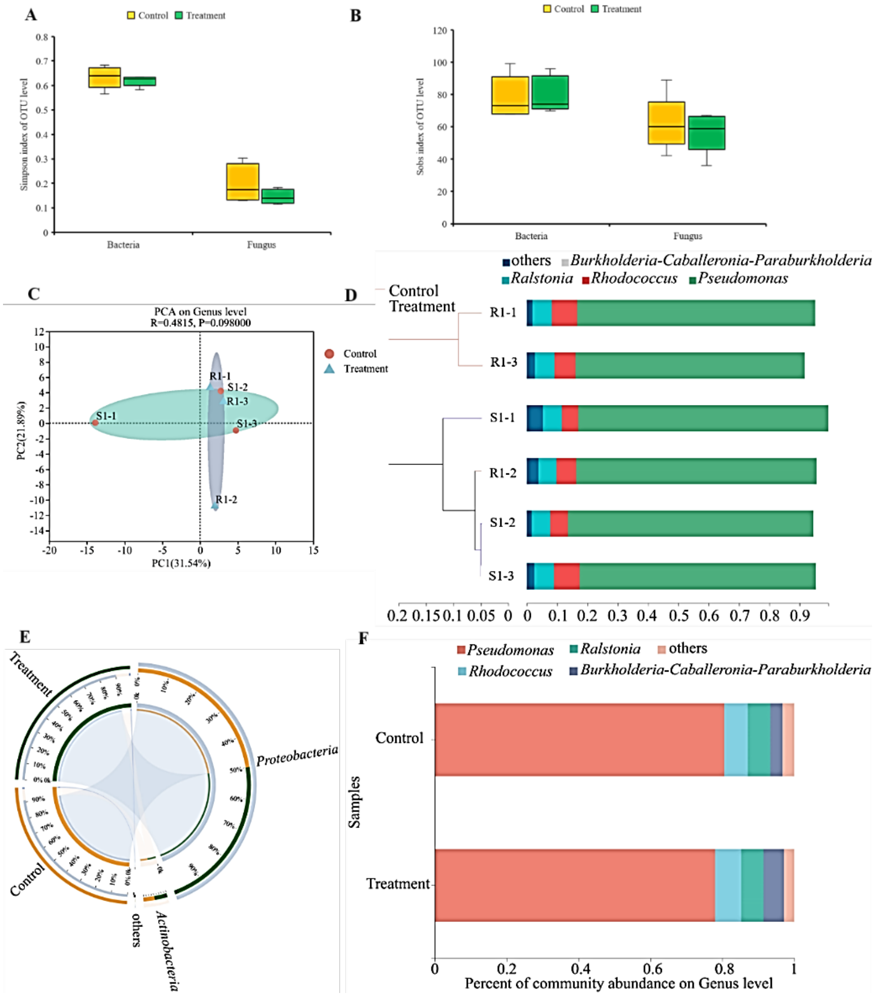

Supplementary Figure 6. The Simpson and sobs of bacterial and fungal in the all samples (A and B) and PCA analysis of bacterial in two groups samples (C) and hierarchical clustering tree of bacterial in two groups samples (D) and circos plot of bacterial composition in two groups samples (E) and the community bar plot analysis of bacterial genus composition in two groups samples (F).

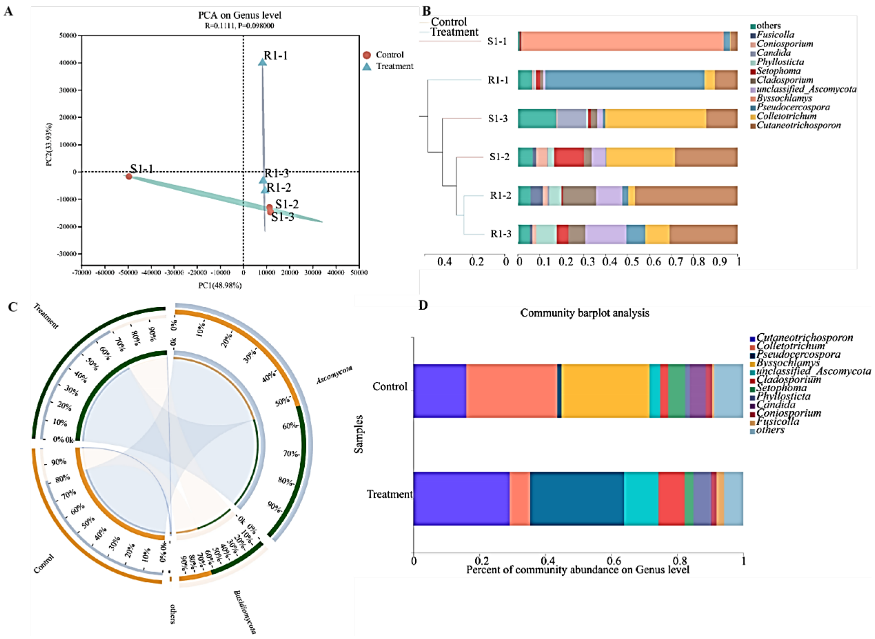

Supplementary Figure 7. PCA analysis of fungal in two groups samples (A) and hierarchical clustering tree of fungal in two groups samples (B) and circos plot of fungal composition in two groups samples (C) and the community bar plot analysis of fungal genus composition in two groups samples (D).

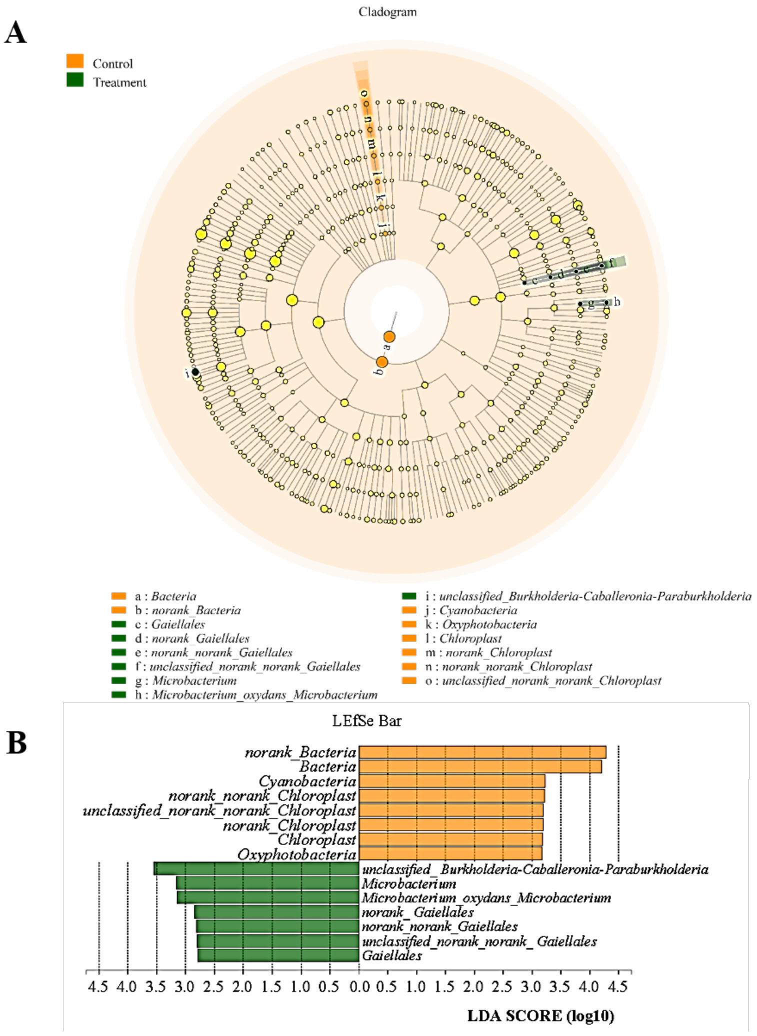

Supplementary Figure 8. Taxonomic cladogram obtained from linear discriminant analysis effect size analysis of 16S sequences

(A) LEfSe cladograms showing the bacterial OTUs enriched in the 'S1' and 'R1' groups. The circles from inner to outer represent the taxonomic levels of phylum, class, order, family, genus, and species. (B) LEfSe-identified LDA bar graphs.

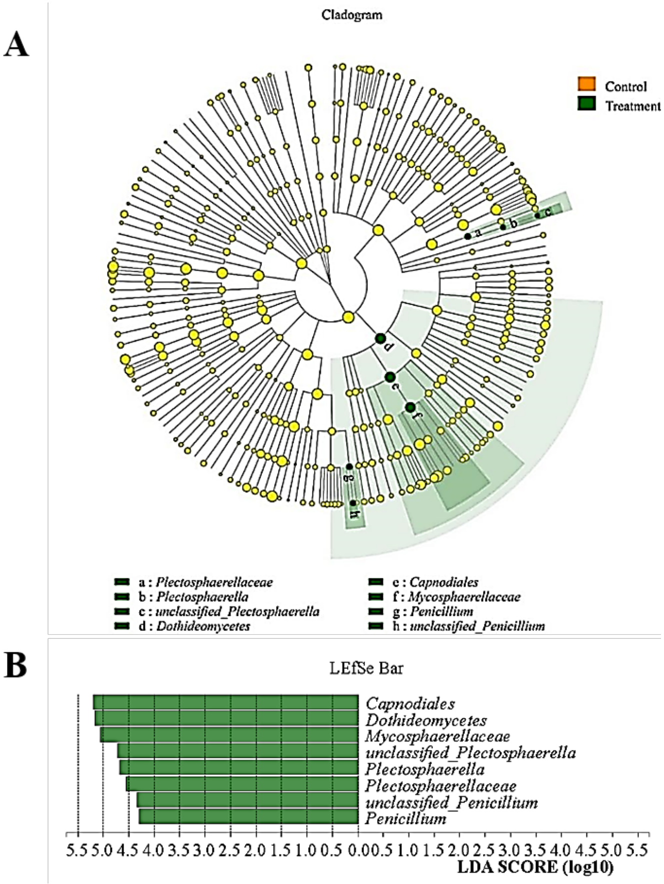

Supplementary Figure 9. Taxonomic cladogram obtained from linear discriminant analysis effect size analysis of ITS sequences

(A) LEfSe cladograms showing the fungal OTUs enriched in the 'S1' and 'R1' groups. The circles from inner to outer represent the taxonomic levels of phylum, class, order, family, genus, and species. (B) LEfSe-identified LDA bar graphs.

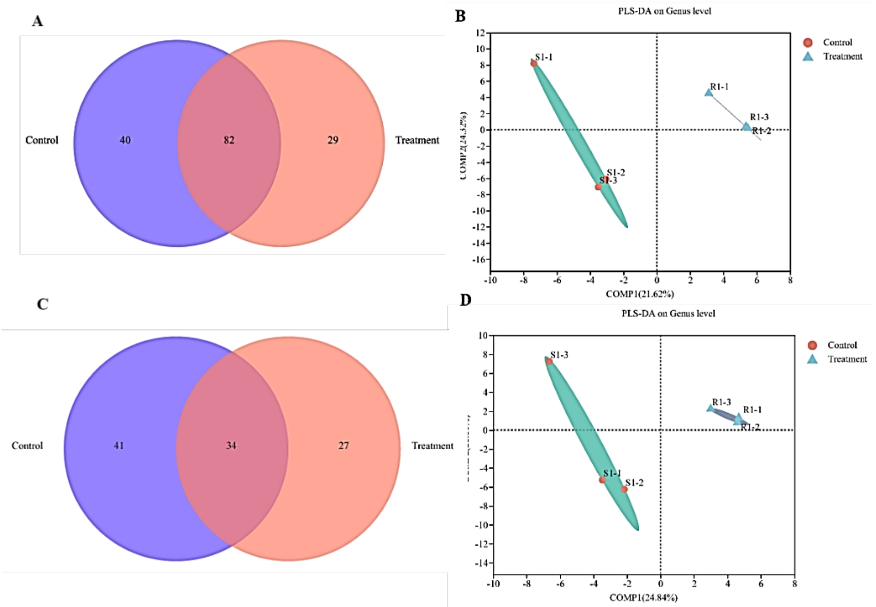

Supplementary Figure 10. Venn plot and PLS-DA of the bacterial and fungal in the two groups samples. (A) Venn plot of bacterial. (B) PLS-DA of bacterial. (C) Venn plot of fungal. (D) PLS-DA of fungal. Control: 'S1' and Treatment: 'R1'.

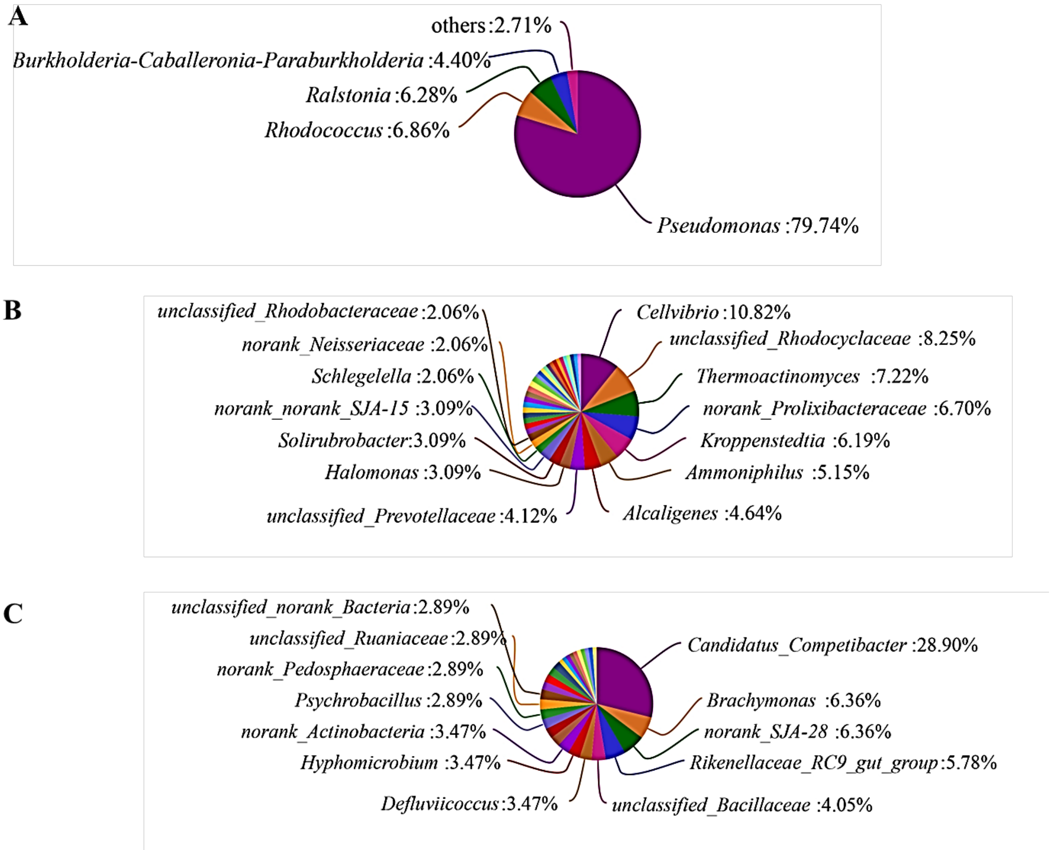

Supplementary Figure 11. Bacterial genera composition in the two tea plant resources. (A) Microbial community pie plot of bacterial in Control and Treatment. (B) Microbial community pie plot of bacterial in control. (C) Microbial community pie plot of bacterial in treatment.

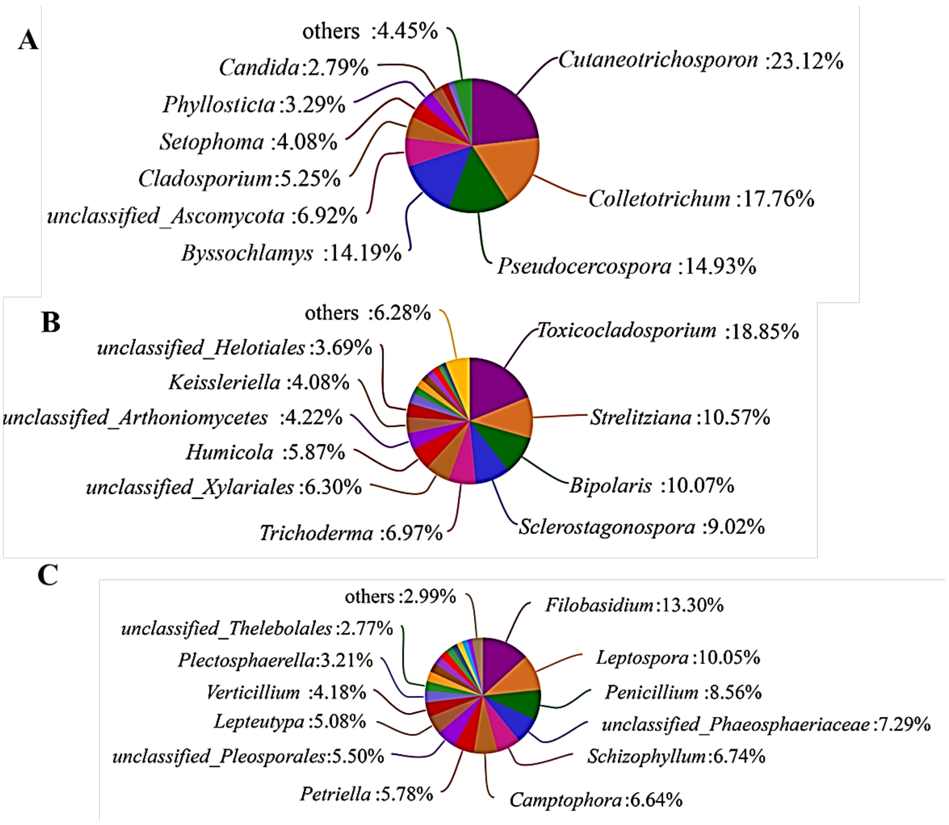

Supplementary Figure 12. The fungal genera composition in the two tea resources. (A) Microbial community pie plot of fungal in Control and Treatment. (B) Microbial community pie plot of fungal in Control. (C) Microbial community pie plot of fungal in Treatment.

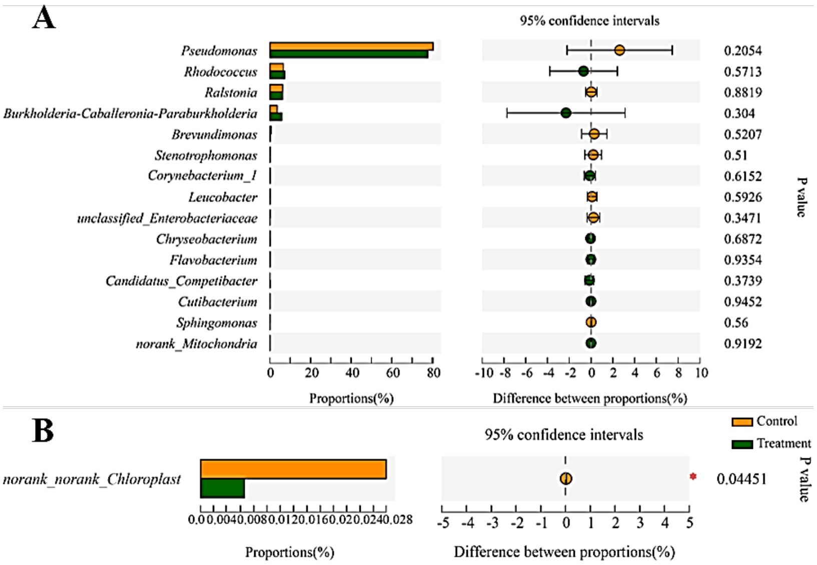

Supplementary Figure 13. The significantly enriched bacterial genera in the "S1" and "R1" samples. (A) The top enriched bacterial genera. (B) The significantly differentially enriched bacterial genera.

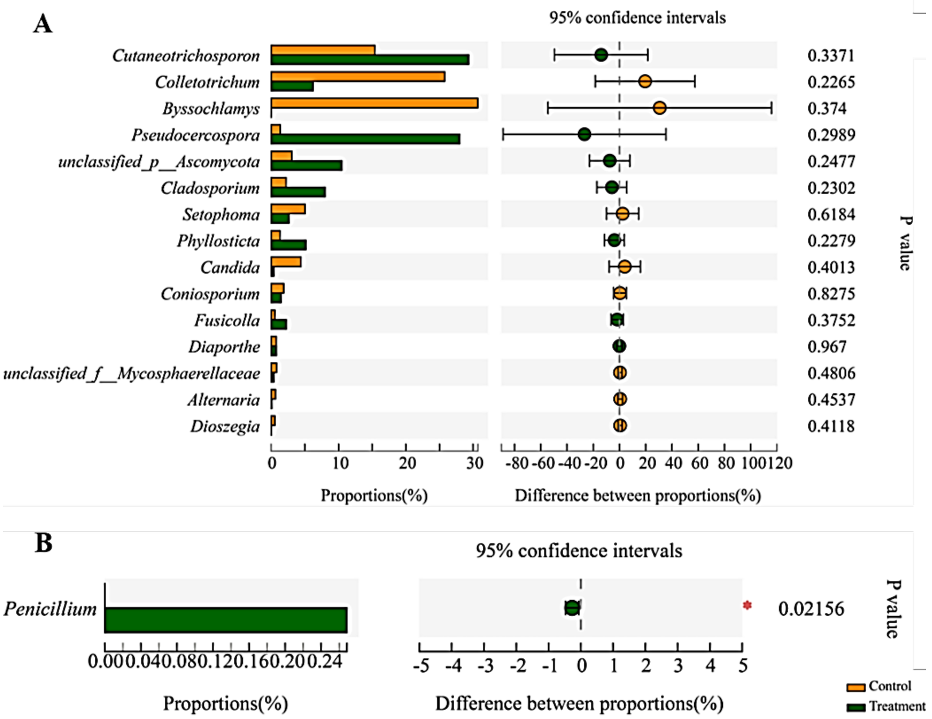

Supplementary Figure 14. The significantly enriched fungal genera in the “S1” and “R1”. (A) The top enriched fungal genera. (B) The significantly differentially enriched fungal genera.

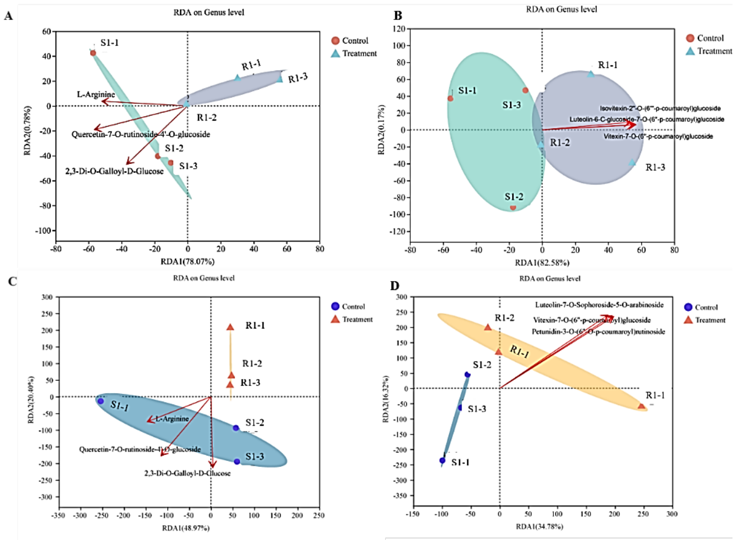

Supplementary Figure 15. Redundancy analysis (RDA) on the differential leaf metabolites contributed the leaf microbiota. (A) RDA on the top three high accumulated SCMs contributed the leaf bacterial genera. (B) RDA on the top three low accumulated SCMs contributed the leaf bacterial genera. (C) RDA on the top three high accumulated SCMs contributed the leaf fungal genera. (D) RDA on the top three low accumulated SCMs contributed the leaf fungal genera.

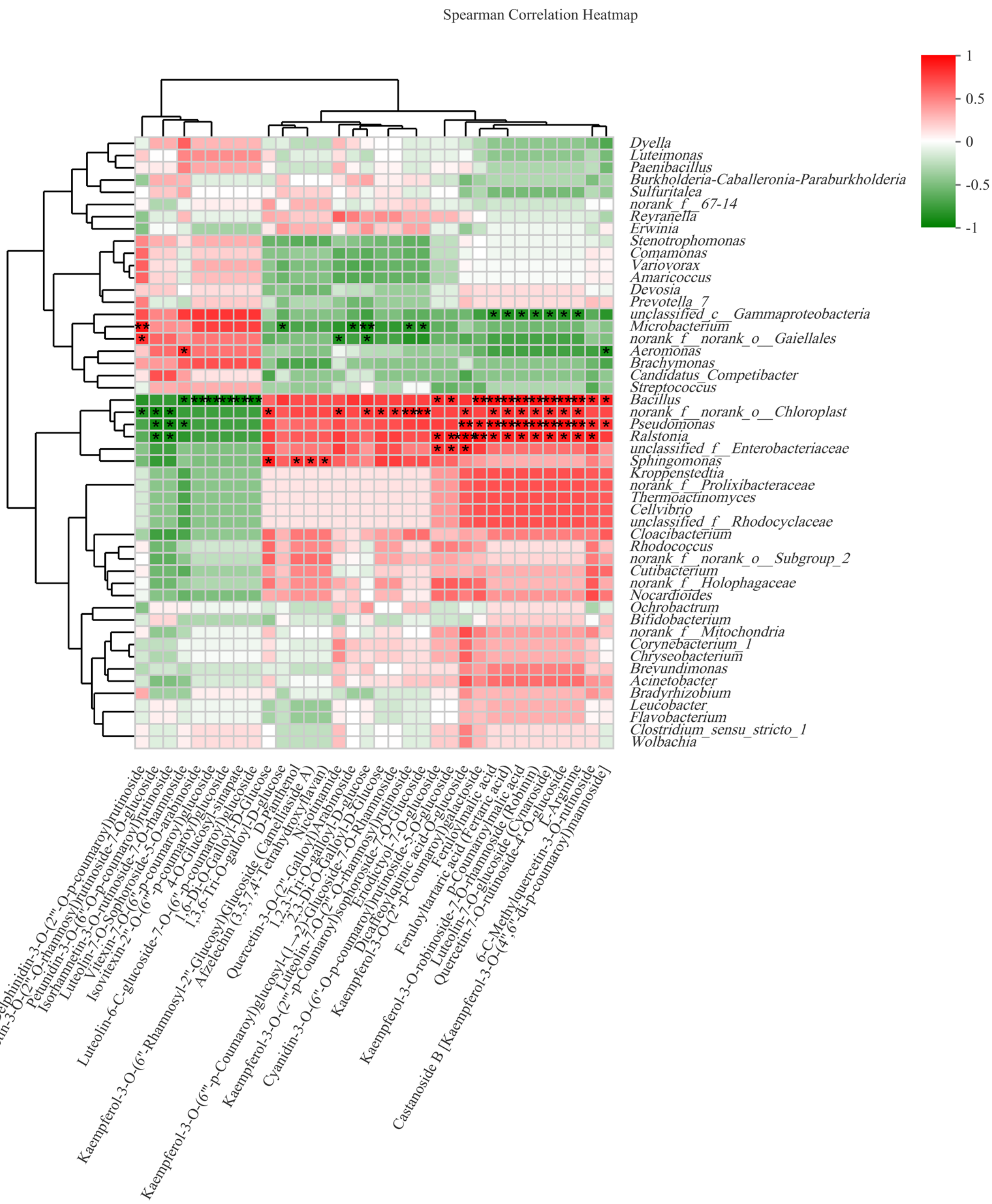

Supplementary Figure 16. Correlation analysis of metabolites and bacterial genus

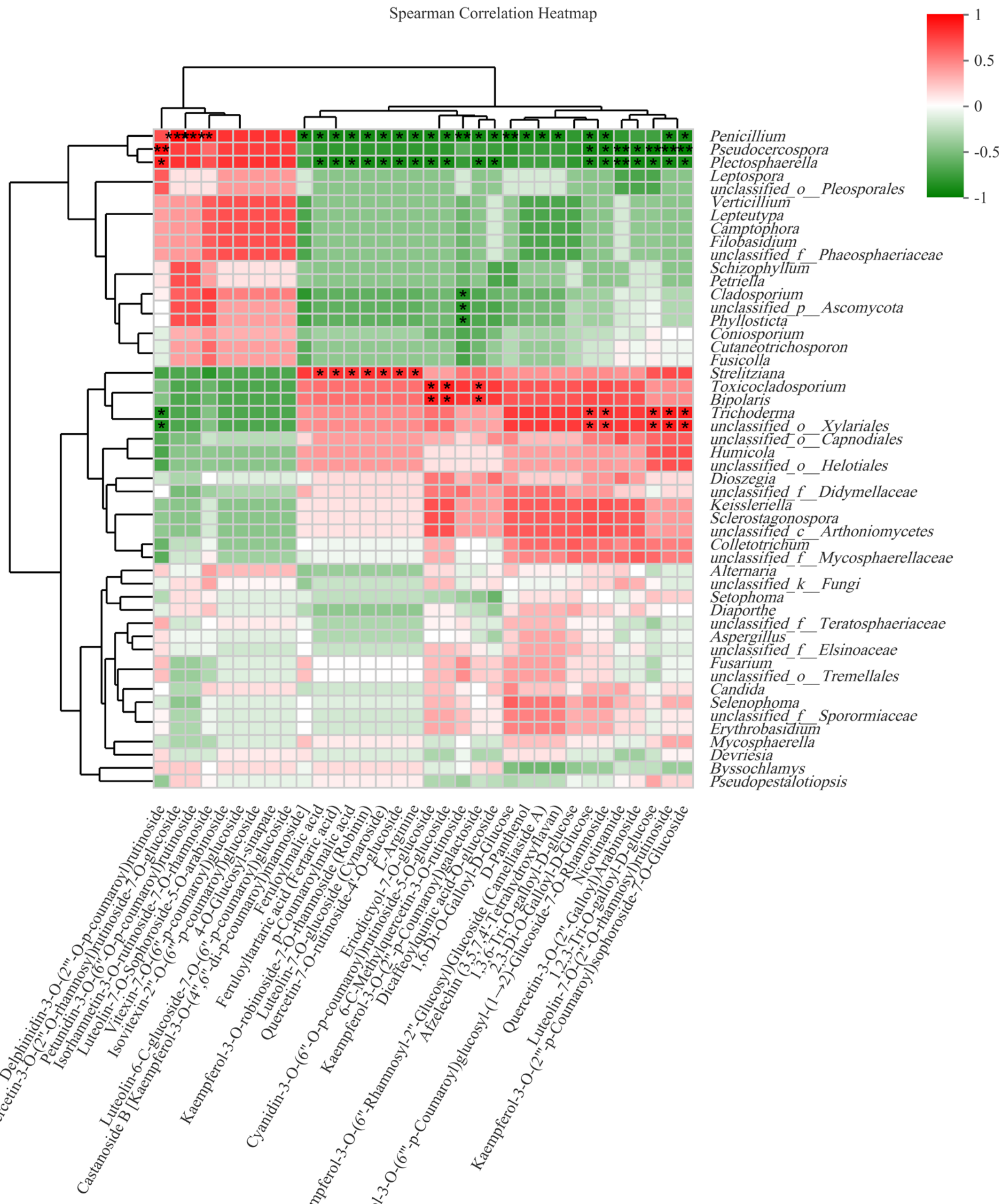

Supplementary Figure 17. Correlation analysis of metabolites and fungal genus
